# Abundance of Pathogenic *Escherichia coli* Virulence-Associated Genes in Well and Borehole Water Used for Domestic Purposes in a Peri-Urban Community of South Africa

**DOI:** 10.3390/ijerph14030320

**Published:** 2017-03-20

**Authors:** Akebe Luther King Abia, Lisa Schaefer, Eunice Ubomba-Jaswa, Wouter Le Roux

**Affiliations:** 1 Departments of Biotechnology, Vaal University of Technology, Private Bag X021, Andries Potgieter Blvd, Vanderbijlpark 1911, South Africa; lutherkinga@yahoo.fr; 2 Natural Resources and the Environment, CSIR, P.O. Box 395, Pretoria 0001, South Africa; lschaefer@csir.co.za (L.S.); eubombajaswa@csir.co.za (E.U.-J.)

**Keywords:** microbial quality, pathogenic *Escherichia coli*, virulence genes, boreholes, wells, public health

## Abstract

In the absence of pipe-borne water, many people in Africa, especially in rural communities, depend on alternative water sources such as wells, boreholes and rivers for household and personal hygiene. Poor maintenance and nearby pit latrines, however, lead to microbial pollution of these sources. We evaluated the abundance of *Escherichia coli* and the prevalence of pathogenic *E. coli* virulence genes in water from wells, boreholes and a river in a South African peri-urban community. Monthly samples were collected between August 2015 and November 2016. In all, 144 water samples were analysed for *E. coli* using the Colilert 18 system. Virulence genes (*eagg*, *eaeA*, *stx1*, *stx2*, *flichH7*, *ST*, *ipaH*, *ibeA*) were investigated using real-time polymerase chain reaction. Mean *E. coli* counts ranged between 0 and 443.1 Most Probable Number (MPN)/100 mL of water sample. Overall, 99.3% of samples were positive for at least one virulence gene studied, with *flicH7* being the most detected gene (81/140; 57.6%) and the *stx2* gene the least detected gene (8/140; 5.7%). Both intestinal and extraintestinal pathogenic *E. coli* genes were detected. The detection of virulence genes in these water sources suggests the presence of potentially pathogenic *E. coli* strains and is a public health concern.

## 1. Introduction

Although the Millennium Development Goals’ (MDGs) target for drinking water supply was globally exceeded, this was not the case in the African region [1]. Many people in Africa, especially in rural communities, still depend on alternative water sources such as rivers [2] and boreholes [3] for household and personal hygiene. Unlike the exceeded target for access to safe drinking water, access to improved sanitation was not met globally, and in the African region, only 7% of the targeted 50% reduction in people without access to improved sanitation was achieved [1]. The lack of sanitation facilities results in the uncontrolled disposal of household and human waste into surrounding water bodies, leading to pollution [4]. In some instances, poorly constructed pit latrines have been used for human waste disposal. However, the construction of these pit latrines uphill from, and in close proximity to, water sources also leads to the pollution of these water sources through leaching [5]. These pollutants could include chemicals such as pharmaceuticals [6] and pathogenic microorganisms [7]. These microorganisms which include bacteria, viruses and parasites, have been found to cause numerous waterborne diseases in humans that use untreated water from such polluted sources [8].

*Escherichia coli* has been extensively studied as an indicator of faecal pollution in water resources, owing to the fact that the organism has been found to colonise the gut of many warm-blooded animals [9]. Though originally considered a commensal, the organism has evolved to include pathogenic strains, causing both intestinal and extraintestinal disease in humans [10,11]. Strains that have been found to cause gastrointestinal illnesses include the enterohaemorrhagic *E. coli* (EHEC), enterotoxigenic *E. coli* (ETEC), enteropathogenic *E. coli* (EPEC), enteroinvasive *E. coli* (EIEC) enteroaggresive *E. coli* (EAEC) and diffusely adherent *E. coli* (DAEC) [12] and they are collectively known as diarrhoeagenic *E. coli*. Those that have been reported to cause extraintestinal illnesses include neonatal meningitis *E. coli* (NMEC) and uropathogenic *E. coli* (UPEC) [13]. Their ability to cause infections in humans lies in a number of virulence traits which the pathogenic forms possess. The virulence genes associated with these intestinal and extraintestinal *E. coli* pathotypes and how they cause disease, have been reviewed previously [13,14].

Groundwater has been recognised as the most abundant and most important source of portable water for human uses around the globe [3]. In Africa in particular, and the world in general, groundwater is considered a relatively safe and cost-effective water source compared to other water sources, especially in those regions where surface water is not readily available [15]. As such, this natural resource is facing constant decrease in quantity and deterioration in quality as a result of both natural and anthropogenic activities, a challenge that is anticipated to get even worse in the 21st century and beyond [16]. Although the limited access to clean portable water has forced many households in South Africa (and many African countries as a whole) to consider alternative sources such as rivers and streams, these sources are usually of poor microbial quality [17,18,19,20]. As such, many other communities have resorted to boreholes and wells to meet their daily water needs. The World Health Organization (WHO) recommends that to ensure good protection of such groundwater sources, they should not be constructed near to or downhill of possible contamination sources such as pit latrines and runoff from animal manure in agricultural areas [8]. However, these recommendations have often been neglected in most communities using groundwater sources, resulting in the pollution of boreholes with chemical and/or pathogenic microorganisms [5]. These pollutants could have adverse health impacts on the lives of people using such polluted water for personal and household hygiene. Currently, there are no clear polices and legislature also governing the use and quality of groundwater in South Africa. While the South African Department of Water and Sanitation is currently drafting a national groundwater strategy [21], there is a need to understand the quality of these water sources so as to ensure protection of the users of the water. Therefore, the current study was carried out to evaluate the microbial quality of wells and boreholes in Stinkwater, a peri-urban community of South Africa, using *E. coli* as an indicator organism. More importantly, the study also sought to determine the prevalence of pathogenic *E. coli* virulence-associated genes in these water sources so as to infer any possibility of infection from the consumption of untreated water from these water sources.

## 2. Materials and Methods

### 2.1. Description of Study Site

The Stinkwater community lies in the northern part of the Gauteng Province of South Africa, close to the border with the Northwest Province. Presently, the Stinkwater community consist of eight extensions comprising a total of 8250 households spread over an estimated surface area of 27.92 km^2^ [22]. Although the main portions of the Stinkwater community consist of subsidised housing, there also exist informal settlements in the area, especially in the outer boundaries of the community [23]. A remarkable characteristic of the Stinkwater community is the lack of access to piped water [24]. As such, the inhabitants are dependent on water delivery from roaming municipal water tankers. As municipal water deliveries are often sporadic, many residents habitually rely on water obtained from hand-dug wells or boreholes in their yards or other communal areas.

### 2.2. Sample Collection and Enumeration of E. coli

**Figure 1:**
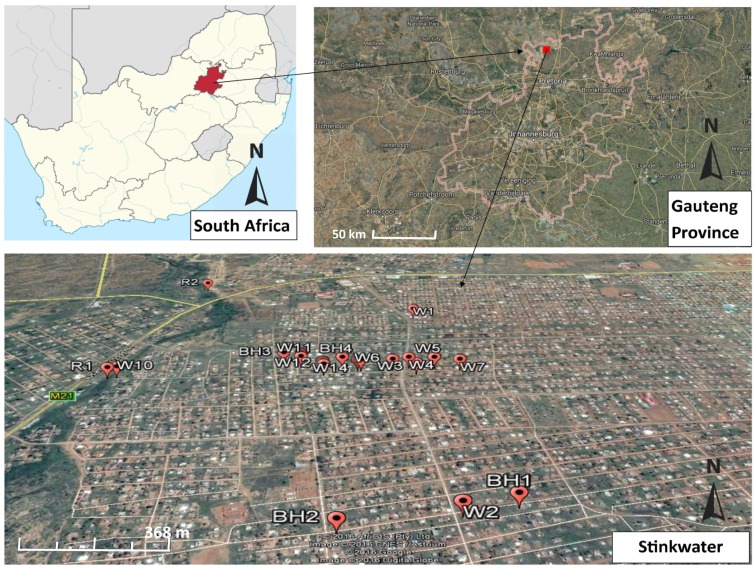
Map of the study area showing sampling points (Source: Google earth). W: well; BH: borehole; R: river (sample collection).

A total of 14 wells (W1–W14), four boreholes (BH1–BH4) and two river sites (R1 and R2) were chosen for the purpose of the current study (Figure 1).

Water samples were collected once a month for a period of 12 months between August 2015 and November 2016 (samples were not collected during the months of December 2015, and January, March and April of 2016). Samples were collected following the South African sampling guide for groundwater [25], transported to the laboratory in cooler boxes containing ice packs and then analysed within 2 h upon arrival at the laboratory. Samples were analysed using the Colilert 18/Quanti-Tray 2000 system (IDEXX Laboratories Inc., Pretoria, South Africa) as previously described [26]. *E. coli* counts were recorded as the Most Probable Number (MPN) per 100 mL (MPN/100 mL) of water sample.

### 2.3. DNA Extraction and Real-Time PCR Identification of E. coli Virulence-Associated Genes

Following 18–24 h incubation of the Quanti-Tray 2000 plates as recommended by the manufacturer, 1 mL of the content of a fluorescent cell on the Quanti-Tray 2000 plates was transferred into 1.5 mL microcentrifuge tubes and DNA was extracted as previously described [27]. The extracted DNA was then used in separate real-time PCR assays for the identification of virulence genes pertaining to different *E. coli* pathotypes. The primers used in each of the PCR assays are given in Table 1.

Sets 2, 3 and 5 PCR were run in multiplex assays while sets 1 and 4 were run in singleplex real-time PCR assays. The multiplex assays were run in total volumes of 20 µL each, while the singleplex assays were run in total volumes of 10 µL each. Assay 2 consisted of 10 µL of 2× SensiFAST High-Resolution Melt (HRM) mix (SF) (Bioline GmbH, Luckenwalde, Germany), at a final concentration of 1×, 0.75 μL each of forward and reverse primers (*eaeA* and *eagg*) at a final concentration of 0.75 μM, 0.2 μL of *ipaH* forward and reverse (final concentration 0.75 μM), 1.6 μL of nuclease free water (NFW) and finally 1 μL of deoxynucleotide (dNTP) Mix (Thermo Fisher Scientific, Edenvale, South Africa) was added to the final overall mixture at a final concentration of 400 μM. Assay 3 consisted of 10 µL SF, 1.5 μL *stx1* (final concentration of 1.5 μM), 0.2 µL *stx2* (final concentration of 0.2 μM), 1.6 μL NFW and 1 μL dNTP Mix. The reaction mixture for Assay 5 was similar to that of Assay 3 with volumes and concentrations of *ST* and *ibeA* being similar to those of *stx1* and *stx2* respectively. Unlike Assays 2, 3 and 5, Assays 1 and 4 were run in a total volume of 10 μL. Assay 1 consisted of 5 µL SF, 0.5 μL *mdh* (forward and reverse; final concentration of 0.5 μM) and 1 μL NFW. The reaction mixture for Assay 4 included 5 µL SF, 0.75 μL *stx2* (forward and reverse; final concentration of 0.75 μM) and 1 μL NFW. For Assays 2, 3 and 5, 5 μL of extracted sample DNA was added while 3 μL of sample DNA was added for Assays 1 and 4. A positive control containing DNA from known *E. coli* strains was included in each of the respective PCR runs. No-template controls, consisting of the respective reaction mixes with NFW (and void of any DNA) added to make up the desired volume, were also included in each assay. All positive controls and PCR cycling conditions were as previously described [27] with a modification of the number of cycles to 45 for all assays.

### 2.4. Data Analysis

The Spearman’s rank correlation was computed to check for any correlation between the abundance of *E. coli* and the overall prevalence of the virulence genes. Data analysis was performed using SPSS 20 (Statistical Package for the Social Sciences; IBM Corporation, Armonk, NY, USA). Before analysis, *E. coli* data was log transformed and statistical tests were considered significant at a 95% confidence limit.

## 3. Results and Discussion

### 3.1. Enumeration of E. coli

A total of 144 samples was collected between August 2015 and November 2016 from the selected 20 sampling sites. Not all the sites were sampled equally as they could not always be accessed routinely due to the unavailability of the owners. The mean MPN of *E. coli* per 100 mL of sample at each site is shown in Table 2. To obtain the mean, all values of <1 were considered as zero while all values of >2419.6 were converted to the nearest whole number (2420).

Due to the vast diversity of microorganisms that can be found in a polluted water source at any given time, it would be extremely laborious and financially demanding to do a complete assessment of the microbial quality of such water. As such, the WHO recommends the use of indicator organisms, of which *E. coli* is the most widely used, for the evaluation of the microbial quality of water [8]. Based on this, the WHO recommends that no *E. coli* be found in any water meant for human consumption [8]. This same limit is adopted by the South African water management authorities [32].

Considering these limits, only one site (BH1) met the WHO and South African guidelines for drinking water quality. However, this cannot be conclusive given that only a single sampling round was analysed during the entire study because of the inaccessibility of the site. Similarly, it cannot be concluded that site W9, BH2 and BH3 were unsafe for consumption given that they were all sampled only once. The high *E. coli* counts observed in samples from these sites could have been the result of a single pollution event and as such, only further sampling of these sites could give a conclusive quality of their water. Nevertheless, consumption of water from these sites on the sampling day could still represent a health risk for household members, especially children and immunocompromised individuals.

The river samples recorded the highest *E. coli* counts during all the sampling days for the entire sampling period (Table 2). This is an indication that the water is constantly polluted. Pollution of this water source could arise from the informal settlements around the neighbouring Soshanguve community through which the river flows, or from the small cattle farms that are present along the banks of the river on the Stinkwater side. Most informal settlements are characterised by the lack of basic sanitation facilities and the inhabitants of such settlements mostly resort to rivers and nearby bushes as their main points of waste disposal. The impact of animal farming [33,34] and informal settlements on the microbial quality of aquatic ecosystems has been previously reported [4,35,36,37,38].

All the wells sampled in this study recorded mean *E. coli* counts above the WHO and the South African recommended limits for drinking water. Most of these wells are shallow, and poorly protected. Rudimentary materials such as zinc or aluminium sheets are typically used to cover the wells and water is extracted with a rope or chain attached to a bucket. These buckets are usually kept under unhygienic conditions on the ground near the well, especially in the communal wells. For example, W1 which recorded the highest mean *E. coli* count of 443.1 MPN/100 mL during the entire study is a communal well located in a public space. Although not used for drinking as indicated by the inhabitants, water from this well is used to wash other household items and motor vehicles. Activities such as motor vehicle washing near water sources has been reported to have negative impacts on water quality [39]. As such, seepage from the nearby pit latrines and the car washing activities around the well could have accounted for the high *E. coli* counts recorded at this site. Similarly, W3 with a mean *E. coli* count of 415.0 MPN/100 mL is located at close proximity to a pit latrine and is used for drinking and other household purposes. Therefore, the poor maintenance of the well, poor hygienic conditions associated with the water extraction process and the close proximity of the well to a pit latrine are factors that could account for the high mean *E. coli* count observed at this site. Such poor hygienic practices have been reported to lead to contamination of groundwater sources [40].

### 3.2. Identification of E. coli Virulence-Associated Genes

A total of 141 fluorescent Quanti-Tray 2000 cells were selected for the identification of the *E. coli* virulence genes. The melt curve analyses of the optimised PCR assays are shown in Figure S1 (Supplementary Materials). Prior to analysing for the virulence genes, all samples were first tested for the presence of the malate dehydrogenase (*mdh*) gene which is a house-keeping gene common to all *E. coli* strains [30]. This was to ensure that all the fluorescence observed was due to the presence of *E. coli*. Of the 141 fluorescent cells analysed, only one cell was negative for the *mdh* gene. This cell was subsequently negative for all other virulence genes tested. Although false positive results have previously been reported with the use of the Colilert system for the identification of *E. coli*, only a single sample (1/141; 0.7%) analysed in the current study was falsely positive compared to the 7.4% to 36.4% reported in other studies [20].

The various virulence genes investigated in the current study were not evenly distributed between the sites (Table 3). As with the abundance of *E. coli*, the Stinkwater River sites recorded the highest number of samples positive for the virulence genes. These sites (R1 and R2) were also the only sites that harboured all the virulence genes (as studied). Virulence genes were not identified in the BH1 sample. Apart from R1 and R2, only W5 was positive for the *stx2* gene of EHEC.

**Figure 2:**
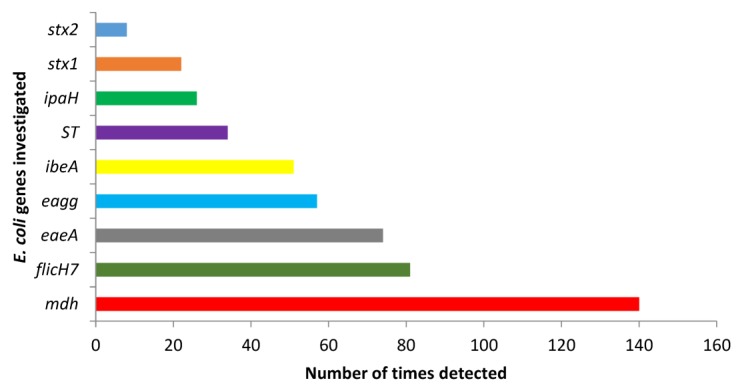
Overall prevalence of the various *E. coli* virulence genes detected during the entire study period.

The overall prevalence of each virulence gene investigated in the current study is shown in Figure 2. Almost all samples (139/140; 99.3%) were positive for at least one of the pathogenic *E. coli* virulence genes investigated in the current study.

There was a positive correlation between the abundance of *E. coli* and the prevalence of the virulence genes (*p* = 0.000; *p* < 0.05) with a correlation coefficient (*r_s_*) of 0.889. This indicates that more virulence genes were identified as the number of *E. coli* increased. The results of the current study contradict the findings of Shelton et al. [41]. In their study, however, Shelton et al., investigated the correlation between the abundance of *E. coli* in a watershed and the presence of only two virulence genes (the intimin (*eae*) and shiga toxin production (*stx*) genes). Like the findings of Shelton et al., but contrary to our findings, Sidhu et al. also reported a lack of correlation between the abundance of *E. coli* and the presence of virulence genes in some sub-tropical surface waters in Brisbane, Australia [42]. Contrary to the approach used in our study, Sidhu et al. worked on pure isolates and this could account for the difference in the correlation between the two studies.

Members of the EHEC group have been globally implicated in numerous bloody diarrhoea outbreaks and haemolytic uremic syndrome [43]. Virulence in these strains is characterised by the presence of the shiga toxin producing genes (*stx1* and *stx2*), the intimin (*eaeA*) gene and other factors such as the *flicH7* gene which codes for the structural flagella antigen in EHEC O157:H7 [44,45]. Strain O157:H7 has been reported to be the most common member of the EHEC group and has been involved in several diarrhoeal disease outbreaks in many developed countries such as the UK, USA, Ireland and Canada [46] as well as many developing countries, including South Africa [47]. In the United States for example, the organism causes an estimated 73,000 cases of illnesses resulting in about 60 deaths every year [48]. The *eaeA* gene is also found in EPEC strains which are mostly responsible for infantile diarrhoea-associated mortality in developing countries and were the first *E. coli* to be associated with human infections [49]. Although diarrhoea caused by EPEC members are usually self-limiting and can easily be managed through oral-rehydration, the onset of the disease is very short, usually 2.9 h following ingestion [50]; complications may arise if the disease is poorly handled, especially in immunocompromised individuals and children. In the current study, all the above genes were detected, with the *flicH7* gene being the most isolated gene (81/140; 57.6%) while the *stx2* gene was the least detected gene (8/140; 5.7%). This indicates that the various water sources in the current study are not only polluted, but also harbour pathogenic forms of *E. coli* with the possibility of adverse health effects to those using the water for drinking and other household purposes without prior treatment. Also, the *stx2* has been isolated in both healthy and diseased cows in South Africa [29], indicating that the high presence of this gene in the current study could have originated from both animal and human sources. The genes responsible for virulence in EIEC (*ipaH*; 18.6%), EAEC (*eagg*; 40.7%) and ETEC (*ST*; 24.3%) were also detected. A study conducted on different surface water bodies in Iran reported that 97% of *E. coli* that was isolated carried the *ipaH* gene [51]. The presence of the invasive plasmid antigen H (*ipaH*) gene in any water body meant for human consumption represents a health risk. Given that the *ipaH* gene (which is also present in *Shigella* spp.) is carried in a plasmid, the gene could easily be transmitted to non-pathogenic *E. coli* strains and other bacterial species, thus transferring the invasive characteristics to the new organisms [51,52]. In a study conducted by Sansonetti et al. [53], the authors reported that loss of the invasive plasmid resulted in the loss of the virulent invasive potential in *Shigella* spp., while the reintroduction of the plasmid into avirulent strains led to the reestablishment of the invasive potentials.

Apart from the genes coding for virulence in the diarrhoeagenic *E. coli*, the gene responsible for virulence in NMEC, an extraintestinal *E. coli*, was also detected. The *ibeA* (invasive brain epithelium A) gene codes for the ability of NMEC strains to invade the meninges in infants, thereby causing meningitis, 10%–30% of which may lead to death [54]. Although these strains are mostly transmitted from mother to child during birth, environmental transmission has also been reported whereby after faecal–oral acquisition, the organism succeeds in crossing the mucosal barrier and then spreads through blood to other organs such as the brain [55,56]. As such, the presence of the *ibeA* gene (51/140; 36.4) in the water from the wells studied indicates that the water contains NMEC strains and is not suitable for consumption, especially in infants. Furthermore, this study adds to the growing evidence that there is a need for clear policies, which can be filtered down to communities, on best practices to protect groundwater sources and to treat and use groundwater. It should be noted, however, that the results presented in the current study are for the prevalence of virulence genes in whole samples and should not be regarded as the abundance of specific *E. coli* pathotypes, given that pure isolates were not obtained prior to detection of the virulence genes.

## 4. Conclusions

The water from the wells and boreholes in the Stinkwater community is not microbiologically safe for human consumption. The high number of samples positive for pathogenic *E. coli* virulence genes indicates that the groundwater is not only faecally polluted, but could also be harbouring pathogenic *E. coli* strains with the potential to cause infection, especially in children and immunocompromised individuals. It is therefore highly recommended that in the absence of clean pipe-borne water, Stinkwater community members be advised to treat the water from these sources before consumption or use in the house.

## Figures and Tables

**Table 1:** Primer sequences used for the identification of *E. coli* virulence-associated genes.

PCR Set	*E. coli* Pathotype	Gene Targeted	Primer Sequence (5′→ 3′)	Reference
1	*E. coli*	*mdh*	F: GGTATGGATCGTTCCGACCT	[28]
			R: GGCAGAATGGTAACACCAGAGT	
2	EPEC/EHEC	*eaeA*	F: ATGCTTAGTGCTGGTTTAGG	[27]
			R: GCCTTCATCATTTCGCTTTC	
	EAEC	*eagg*	F: AGACTCTGGCGAAAGACTGTATC	
			R: ATGGCTGTCTAATAGATGAGAAC	
	EIEC	*ipaH*	F: GTTCCTTGACCGCCTTTCCGATACCGTC	
			R: GCCGGTCAGCCACCCTCTGAGAGTAC	
3	EHEC	*stx1*	F: CTGGATTTAATGTCGCATAGTG	
			R: AGAACGCCCACTGAGATCATC	
		*flicH7*	F: TACCATCGCAAAAGCAAC TCC	[29]
			R: GTCGGCAACGTTAGTGATACC	
4	EHEC	*stx2*	F: CCATGACAACGGACAGCAGTTCCT	[30]
			R: GTCAACTGAGCACTTTG	
5	ETEC/NMEC	*ST*	F: TTTCCCCTCTTTTAGTCAGTCAACTG	
			R: GGCAGGATTACAACAAAGTTCACA	
		*ibeA*	F: TGGAACCCCGCT CGTAATATAC	[31]
			R: CTGCCTGTTCAAGCATTGCA	

**Table 2:** Mean *E. coli* count (Most Probable Number per 100 mL; MPN/100 mL) per sampling site.

Site	n	Mean	Min	Max
W1	12	443.1	<1	>2419.6
W2	12	51.6	<1	307.6
W3	12	415.0	<1	1986.3
W4	12	41.7	<1	325.5
W5	12	250.1	<1	>2419.6
W6	12	174.7	<1	1553.1
W7	11	149.7	12.1	365.4
W8	12	214.2	<1	>2419.6
W9	1	44.8 *	44.8	44.8
W10	4	24.23	3	59.8
W11	2	1.5	1	2
W12	5	2.36	<1	9.8
W13	2	26.45	13	39.9
W14	4	7.875	<1	27.5
BH1	1	0 *	<1	<1
BH2	1	40.4 *	40.4	40.4
BH3	1	16.6 *	16.6	16.6
BH4	5	3.2	<1	12
R1	11	1119.9	43.9	>2419.6
R2	12	1147.9	178.5	>2419.6

* Values represent results of a single sampling round and should not be regarded as means.

**Table 3:** Number of samples positive for each gene per sample site.

Site	n	*eaeA*	*eagg*	*ipaH*	*ST*	*ibeA*	*stx1*	*stx2*	*flicH7*
W1	12	7	3	2	1	6	1	0	8
W2	12	2	5	3	1	1	1	0	5
W3	12	6	6	2	3	5	1	0	8
W4	12	7	2	2	3	4	1	0	6
W5	12	5	4	0	1	3	1	1	4
W6	12	7	6	3	2	3	2	0	6
W7	11	6	4	0	2	4	0	0	8
W8	12	4	2	2	6	1	1	0	4
W9	1	1	1	0	0	0	0	0	1
W10	4	2	1	1	0	2	1	0	3
W11	2	1	0	0	0	1	0	0	0
W12	5	1	1	1	0	0	0	0	1
W13	2	2	1	2	0	0	0	0	2
W14	4	0	0	3	0	2	0	0	1
BH1	1	0	0	0	0	0	0	0	0
BH2	1	1	0	0	0	1	1	0	1
BH4	1	0	1	1	0	0	0	0	0
R1	11	11	10	1	7	8	5	3	11
R2	12	11	10	3	8	10	7	4	12

## Supplementary Materials

The following is available online at www.mdpi.com/1660-4601/14/3/320/s1, Figure S1: High-resolution melt curves (HRM) for each PCR assay.
