# Supplementary material for: Abundance of Pathogenic Escherichia coli Virulence-Associated Genes in Well and Borehole Water Used for Domestic Purposes in a Peri-Urban Community of South Africa
Source: Int J Environ Res Public Health. 2017 Mar 20;14(3):320. doi: 10.3390/ijerph14030320 (PMC5369156; doi:10.3390/ijerph14030320)
Supplement: Supplementary file 1 [file ijerph-14-00320-s001.pdf]

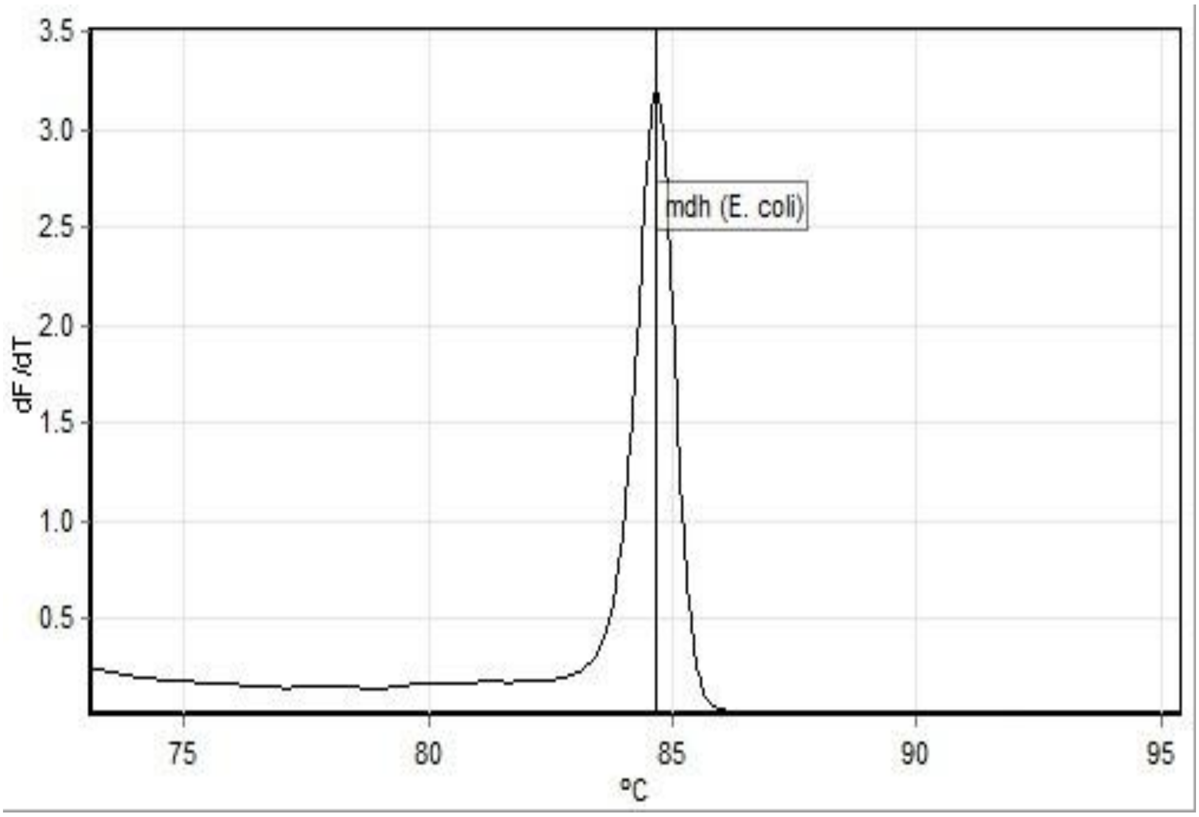
3.5
3.0
2.5
2.0
1.5
1.0
0.5
dF /dT
mdh (E. coli)
75
80
85
90
95
°C

a

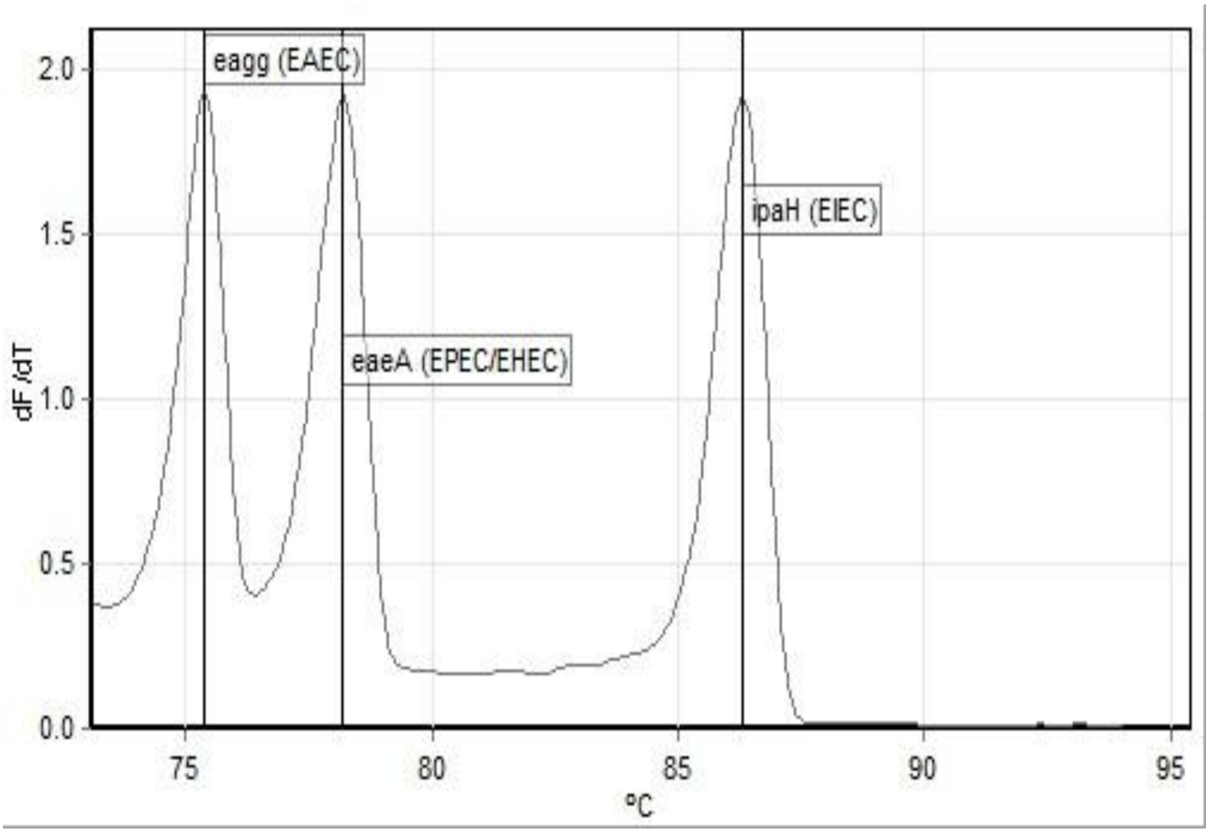
2.0
1.5
1.0
0.5
0.0
dF /dT
eagg (EAEC)
eaeA (EPEC/EHEC)
ipaH (EIEC)
75
80
85
90
95
°C

b

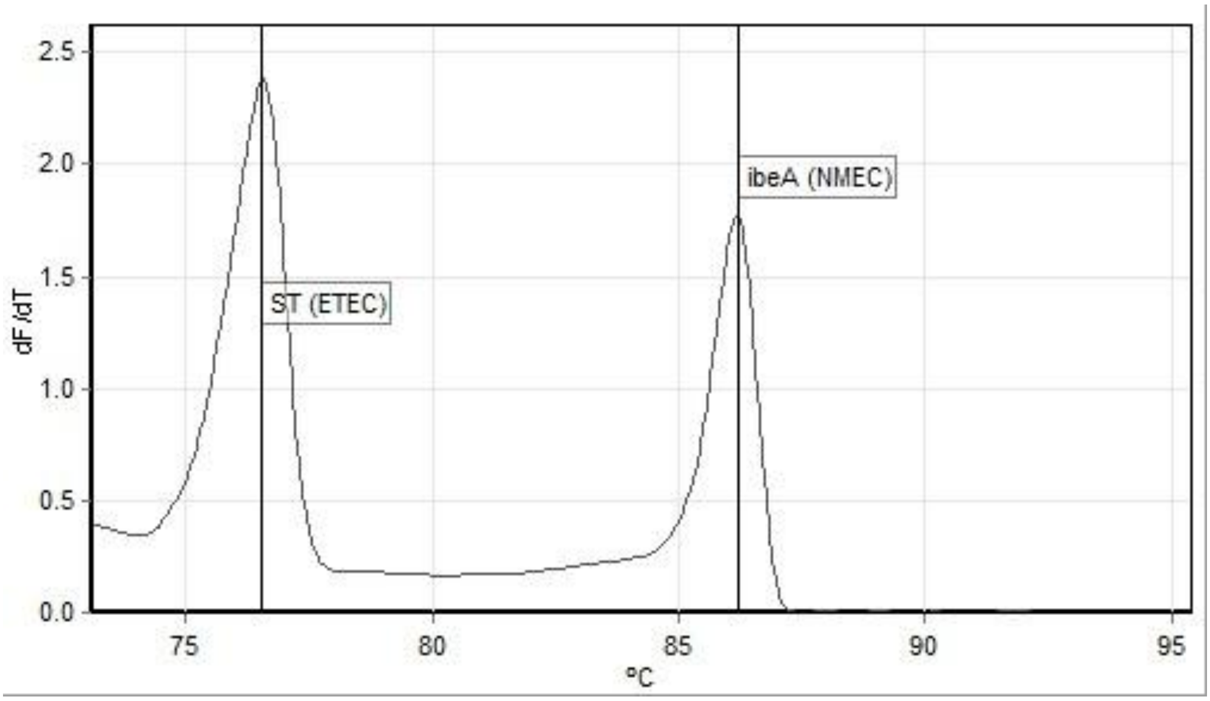
2.5
2.0
1.5
1.0
0.5
0.0
dF /dT
ST (ETEC)
ibeA (NMEC)
75
80
85
90
95
°C

c

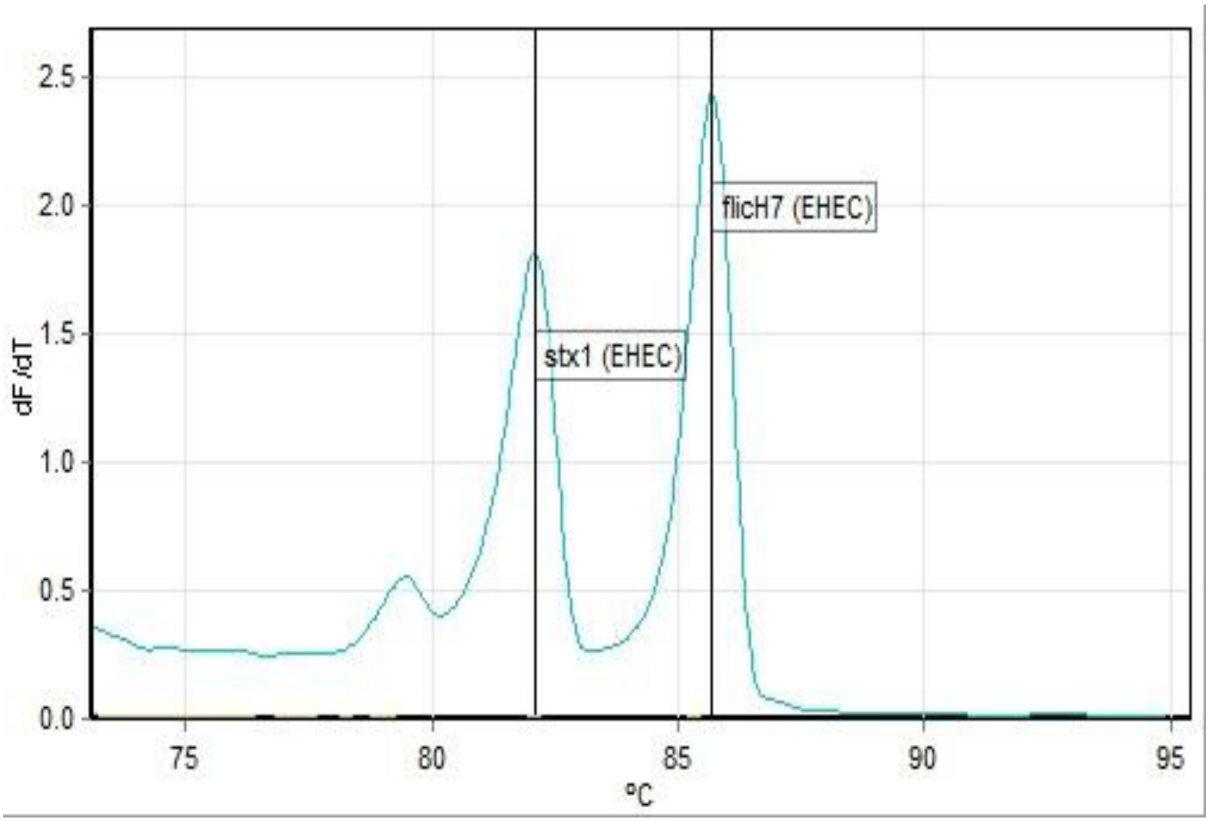
2.5
2.0
1.5
1.0
0.5
0.0
dF /dT
stx1 (EHEC)
flicH7 (EHEC)
75
80
85
90
95
°C

d

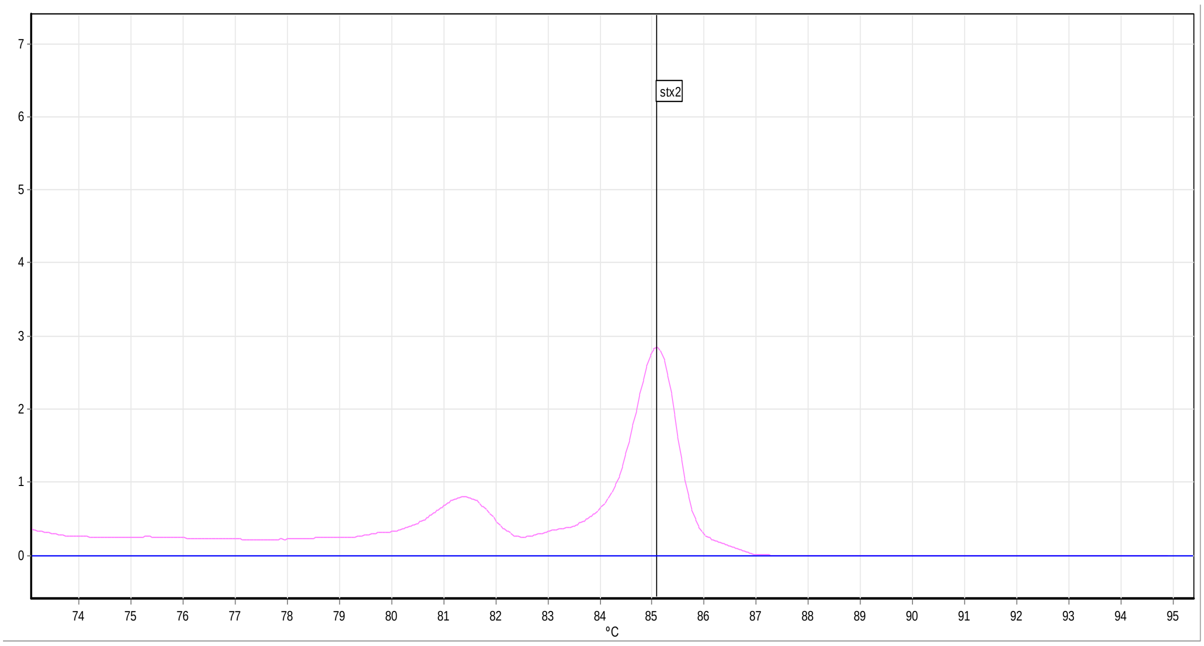

e

Figure S1. High-resolution melt curves (HRM) for each PCR assay. a = *mdh* (E. coli); b = *eagg*/*eaeA*/*ipah* (EHEC/EAEC/EIEC); c = *ST*/*ibeA* (ETEC/NMEC); d = *stx1*/*flicH7* (EHEC); e = *stx2* (EHEC)
